# Preparation and Characterization of a Novel Activated Carbon@Polyindole Composite for the Effective Removal of Ionic Dye from Water

**DOI:** 10.3390/polym14010003

**Published:** 2021-12-21

**Authors:** Bushra Begum, Saba Ijaz, Rozina Khattak, Raina Aman Qazi, Muhammad Sufaid Khan, Khaled H. Mahmoud

**Affiliations:** 1 Department of Chemistry, Shaheed Benazir Bhutto Women University, Peshawar 25000, Pakistan; sabaijaz240@gmail.com (S.I.); rainaaman@sbbwu.edu.pk (R.A.Q.); 2 National Centre of Excellence in Physical Chemistry, University of Peshawar, Peshawar 25120, Pakistan; 3 Department of Chemistry, University of Malakand, Chakdara 18800, Pakistan; sufaidkhan1984@uom.edu.pk; 4 Department of Physics, College of Khurma University College, Taif University, Taif 21944, Saudi Arabia; k.hussein@tu.edu.sa

**Keywords:** conducting polymer composites, polyindole, activated carbon, adsorption, Malachite Green, wastewater treatment

## Abstract

The present study is aimed at the synthesis and exploring the efficiency of a novel activated carbon incorporated polyindole (AC@PIN) composite for adsorptive removal of Malachite Green (MG) dye from aqueous solution. An AC@PIN hybrid material was prepared by in situ chemical oxidative polymerization. The physico-chemical characteristics of the AC@PIN composite were assessed using Fourier-transform infrared spectrometer, X-ray diffraction, scanning electron microscopy, energy dispersive X-ray spectroscopy, ultraviolet visible spectroscopy, and determination of point of zero charge (pH_PZC_). A series of adsorption studies was conducted to evaluate the influence of operational parameters such as pH, contact time, initial dye concentration, AC@PIN dosage, and temperature on dye adsorption behavior of developed composite. A maximum dye removal percentage (97.3%) was achieved at the pH = 10, AC@PIN dosage = 6.0 mg, initial dye concentration 150 mg L^−1^, and temperature = 20 °C. The kinetic studies demonstrated that the adsorption of MG on AC@PIN followed pseudo-second-order model (R^2^ ≥ 0.99). Meanwhile, Langmuir isotherm model was founded to be the best isotherm model to describe the adsorption process. Finally, the recyclability test revealed that the composite exhibits good recycle efficiency and is stable after 5 cycles. The obtained results suggest that AC@PIN composite could be a potential candidate for the removal of MG from wastewater.

## 1. Introduction

Designing a novel and cost-efficient material for the purification of wastewater is one of the hottest topic of research across the world. The roots cause of water contamination is industrial effluents, agriculture, and nuclear waste [1,2]. Effluents from textile, cosmetic, paint, printing, leather, and plastic industry, primarily contain a complex range of colored chemical substances and heavy metals. However, dye is the major culprit in water pollution. It is estimated that about 40,000 tons dyes end up in wastewater streams, annually [1,3,4]. The presences of these non-biodegradable dyes in small amount can drastically cause carcinogenicity, toxicity, and inhibit photosynthesis of aquatic plants as there is reduced penetration of dissolved oxygen and light in water [1,2,5]. Therefore, the removal of dyes from wastewater through an economic way is highly desirable [1].

Literature reports numerous physico-chemical and biochemical techniques like ozonation, ion exchange, photocatalytic degradation, membrane separation, coagulation, adsorption, and electrochemical methods have been practiced for the elimination of noxious organic dyes from water streams [1,2,3,4,5]. Among these, adsorption is the most commonly applicable, simple, and economically viable method for the treatment of wastewater. Besides, it is a convenient, efficient, and eco-friendly method for the removal of colorants [2,3,5]. The operation cost of adsorption process is mainly dependent upon the adsorbent material [1]. Ample of reports are available in literature on employing adsorbents such as biochar, chitosan, zeolite, activated carbon (AC), rice husk, clay, orange peel, polymers, and pine bark for water purification [1,4,5,6]. However, flammability, weak hydrophilic nature, and difficulty in regeneration and processability are the major constraints in the wide applicability of the already reported adsorptive material [3,5].

Conjugated polymers like polyaniline (PANI), polypyrrole (PPy), etc., and their composites with various organic and inorganic materials have gained substantial attention of research in many applications, particularly in wastewater purification [1,3,4,5]. The attractive features like structural porosity, improved mechanical stability, regenerability, selectivity, and good adsorption capacity make polymer-based adsorbents as potential candidates for water decontamination. In addition, they are inexpensive, easy to synthesize, biodegradable, and environmental benign in nature [1,4,5]. Various researchers reported the potential applicability of conjugated polymers and their composites for the adsorption of noxious dyes from waste water. For instance, Abdelghani et al. [1] prepared PANI@Almond shell bionanocomposite through in situ interfacial oxidation method and used for the adsorptive removal of Orang G dye from aqueous solution. The material showed 190.98 mg g^−1^ as maximum adsorption capacity. Similarly, Sudhindra et al. [5] prepared PANI-multi-walled carbon nanotubes composite and reported its ability as an efficient and reusable adsorbent for Methyl Orange dye from water. Mudassir et al. [7] reported that PANI@AC could be a potential adsorbent for Methyl Orange removal. Sarojini and co-worker [8] evaluated the performance of PPy-marine biomass biocomposite for the elimination of Malachite Green from wastewater. Moreover, Saima and co-researchers [9] prepared different polymeric biocomposites based on PANI, PPy, starch, chitosan, and sugarcane bagasse and successfully employed as adsorbents for the Acid Black-234 under various condition. Though, these polymeric composites are environmentally friendly, inexpensive, and effective for the decontamination of water from dyes, still there is a need for the other novel, upgraded, and efficient adsorbent materials with enhanced adsorption capacity and processability in water treatment process.

**Scheme 1:**
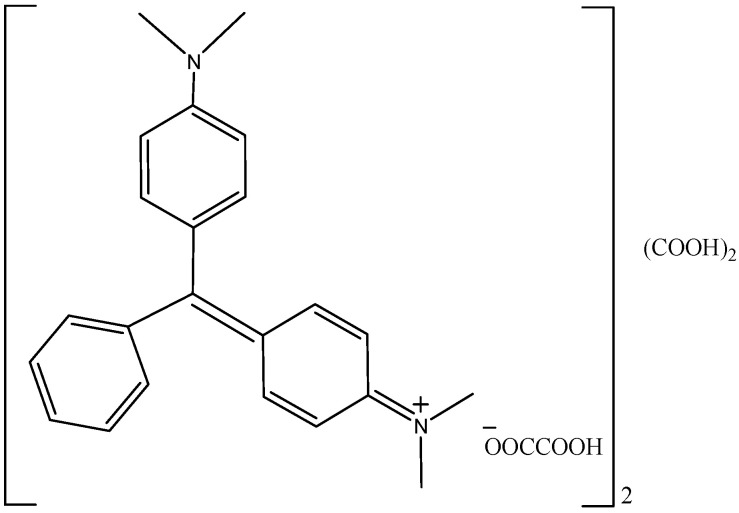
Molecular structure of Malachite Green dye.

Polyindole (PIN), a newly emerging conjugated polymer and its composites, have found significant importance in many fields such as sensors, supercapacitors, batteries, anticorrosion, etc. The major reasons for its applicability are its excellent mechanical stability, reasonably good electro-activity, optical properties, etc. [10,11]. In addition, PIN-based materials have been employed for adsorptive removal of heavy metals from wastewater. For example, PIN nanofibers were used for adsorption of copper (II) by Zhijiang et al. [12]. Similarly, Cai, Z. et al. [13,14] prepared surface-functionalized PIN nanofibers for effective removal of Cr(VI), Pb(II), and Cd(II) from aqueous solutions. Besides, polyacrylonitrile/PIN composite and zinc oxide/magnesium oxide/PIN have been used for Cu(II) and Pb(II), respectively [15,16]. However, literature reveals no study on the usage of PIN and its composites for the removal of dye from water. Thus, it is hoped to obtain new polymeric composite in this work by merging PIN with AC that could not only upgrade the physical and chemical features of the composite for many physico-chemical processes, especially, dye adsorption, but also process the sustainability at a low cost. Based on the abovementioned facts, the present study was carried out to successfully synthesize a novel AC integrated PIN (AC@PIN) hybrid material for the first time, as far as we know, and investigate its efficacy for the removal of ionic dye, Malachite Green (MG), from the aqueous system (Scheme 1). The AC@PIN composite was prepared by a simple and scalable one-pot in situ chemical polymerization method. The MG adsorption efficiency of the prepared AC@PIN composite in terms of different experimental conditions including pH, temperature, initial dye concentration, AC@PIN dosage, and contact time was evaluated. In addition, isotherm and kinetic studies of the MG adsorption on AC@PIN were performed by applying various mathematical models to the experimental data.

## 2. Materials and Methods

### 2.1. Chemical and Reagents

The indole (C_8_H_7_N, >99%), sodium dodecyl sulfate (CH_3_(CH_2_)_11_OSO_3_Na, >95%), benzoyl peroxide (C_14_H_10_O_4_, >98%), Malachite Green (C_23_H_25_N_2_·C_2_HO_4_·0.5C_2_H_2_O_4_, >99%), sodium hydroxide (NaOH, >99.8%), hydrochloric acid (HCl, 37%), and activated carbon (AC, 98%) were purchased from Sigma Aldrich (St. Louis, MO, USA). Chloroform (CHCl_3_, >99%) and ethanol (C_2_H_5_OH, >99%) were obtained from BDH(Poole, UK). Phosphoric acid (H_3_PO_4_, 85%) and perchloric acid (HClO_4_, >70%) used in the experiment were borrowed from Scharlau (Barcelona, CAT, Spain). Malachite Green stock solution (1 g L^−1^) was prepared by dissolving an accurately weighed amount of dye in deionized water, and the desired working concentrations were prepared using the stock solution. All these research grade chemicals were used as received without any further processing.

### 2.2. Chemical Activation of AC

Commercially available AC was chemically activated using 30% *w*/*v* H_3_PO_4_ solution. In context to this, AC was mixed with H_3_PO_4_ solution to make slurry of 1:1. The mixture was stirred for about 2–3 h to make it uniform. The slurry was dried in an oven at 120 °C for 6 h and then cooled to room temperature. Subsequently, the mixture was washed thoroughly with double distilled water to remove extra phosphate ions and then dried again in an oven at 100 °C for 24 h to remove moisture.

### 2.3. Synthesis of AC@PIN Composite

**Scheme 2:**
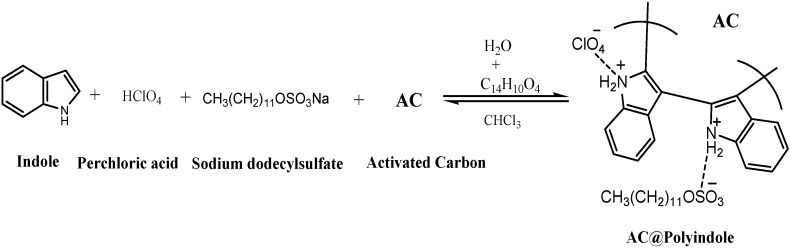
Probable polymerization mechanism of AC@PIN.

AC@PIN composite was synthesized via in situ chemical oxidative polymerization using varying amount of AC. The process of synthesis was executed by gradual addition of 0.644 g of benzoyl peroxide into 250 mL beaker loaded with 30 mL of chloroform while being on constant mechanical stirring. Subsequently, 0.020 g of sodium dodecylsulfate followed by AC (0.01–0.3 g) dispersed in 1 M HClO_4_ (30 mL) was slowly added to above oxidizing mixture. Afterwards, 0.249 g of indole was introduced into reaction flask. Constant mechanical stirring for about 24 h at room temperature yielded a greenish colored product. Around 5–10 mL of ethanol was poured to stop the reaction. The polymer precipitates were then filtered and washed with ethanol until a light yellowish filtrate was obtained and it was then dried. The possible polymerization mechanism is presented in Scheme 2.

A series of experiments were carried out with varying amount of AC, i.e., 0.01 g (AC@PIN-1), 0.10 g (AC@PIN-2), 0.25 g (AC@PIN-3), and 0.30 g (AC@PIN-4) via keeping concentration of all other components constant.

For comparison, pristine polymer (PIN) without AC was also synthesized using same chemical polymerization method.

### 2.4. Characterizations

The functional group analysis and molecular structures of PIN based polymers were evaluated by Fourier Transform Infra-Red (FTIR) spectrometer (Schimadzu IR Prestige-21, Duisburg, Germany), in the range of 400–4000 cm^−^^1^. The polymer crystallinity was investigated by using X-ray diffractometer (XRD) (Malvern Panalytical, Malvern, UK) technique equipped with (Cu-Kα) radiations source in 2theta range of 10–80°. Scanning Electron Microscopy (SEM) (TESCAN Vega 3, Brno, Czechia) was used for morphological studies, whereas Energy Dispersive X-ray (EDX) (Vario EL CHNS, ElementarAnalysensysteme GmbH, Hanau, Germany) spectroscopic analysis was carried out to examine the elemental composition of prepared polymer composites. The optical absorption properties were studied by using UV-Vis spectrophotometer (UV-1800, Schimadzu, Kyoto, Japan) and spectra obtained are recorded in the range of 200–800 nm in ethanol.

The point of zero charge (pH_PZC_) of AC@PIN composite was obtained by the solid addition method using 0.1 M NaCl solution [17]. Assays were performed in a series of glass vials, each containing 20 mL of 0.1 M NaCl solution. The initial pH (pH_i_) was adjusted using either 0.1 M NaOH or 0.1 M HCl solution. Then, 0.002 g of AC@PIN was added to each vial and the suspension was stirred at 20 °C temperature for 24 h with continuous shaking (@300 RPM). The final pH (pH_f_) of each suspension was recorded and pH_PZC_ was obtained from the graph between ∆pH verses pH_i_.

### 2.5. Adsorption Studies

Initially, we tested PIN, AC, and AC@PIN composite for the removal of MG. For each prepared adsorbent, the adsorption study was performed using a known amount of adsorbent in contact with 20 mL of the MG solution at neutral pH and at room temperature.

The adsorption behavior of MG onto the AC@PIN composite was analyzed via Batch mode studies under various experimental conditions such as solution pH (from 2 to 10), AC@PIN dosage (from 6.0 to 8.0 mg), adsorption contact time (from 0 to 60 min), MG initial concentration (from 50 to 200 mg L^−^^1^), and temperature (from 20 to 55 °C). The influence of the operational parameters on the adsorption efficiency of AC@PIN was evaluated by varying one variable at a time. The pH was adjusted by adding an appropriate volume of NaOH (0.1 M) or HCl (0.1 M) solutions. The concentration of MG before and after adsorption was determined from the calibration curve of a series of working concentrations of MG according to Beer-Lambert’s law at the pre-determined λ_max_ (614 nm). The absorbance values (at 614 nm) of each solution were measured from UV-Vis spectra recorded by a UV-Vis spectrophotometer (Schimadzu, UV-1800). Afterwards, the MG adsorption onto AC@PIN composite was confirmed by separating the adsorbent from the MG solution after adsorption. The dye adsorbed capacity (Q_t_, mg g^−^^1^) and percent removal efficiency (R, %) were calculated using Equations (1) and (2), respectively [5,6].

Qt = [(C_o_ − C_t_)/m] × V (1)

R = [(C_o_ − C_t_)/C_o_] × 100 (2)

where C_o_ (mg L^−^^1^) is initial dye concentration, C_t_ (mg L^−^^1^) is the dye concentration at any time, V (L) is the volume of solution, and m (g) is the mass of the adsorbent.

The adsorbent reusability was investigated by the adsorption and desorption of MG loaded AC@PIN composite. The regenerated AC@PIN was filtered, washed repeatedly with deionized water, dried, and then reactivated using 1 M HClO_4_ for 2 h at 20 °C under constant stirring. The desorbed AC@PIN was again used for the MG adsorption under optimized operating conditions. This process was repeated for five cycles.

## 3. Results and Discussion

### 3.1. Chemical Structure Analysis

**Figure 1:**
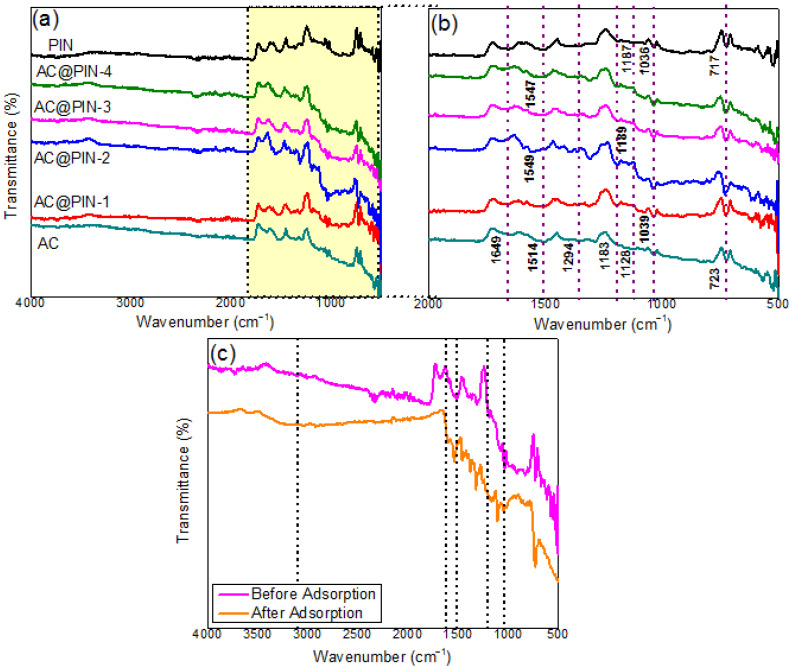
FTIR spectra of (**a**) PIN, AC, and AC loaded various AC@PIN composites, (**b**) re-scaled spectra from 2000 to 500 cm^−^^1^, and (**c**) comparative spectra of AC@PIN-3 before and after adsorption of MG dye.

The FTIR spectra of pristine PIN, AC, and AC@PIN composites synthesized via chemical route are presented in Figure 1a,b.The FTIR spectrum of PIN displays sharp bands located at 717 cm^−^^1^, 1417 cm^−^^1^ and 1513 cm^−^^1^ indicates out of plane deformation of C–H bond in benzene ring, stretching mode of C–N, and indole, respectively [18,19]. The incorporation of ClO_4_^−^ from HClO_4_ in PIN is indicated by Cl–O stretching peak at 1036 cm^−1^ [20]. The bands located at 1589 cm^−^^1^ and 1187 cm^−^^1^ were assigned to C=C vibration mode of aromatic ring and asymmetric stretching of S=O that originates from SO^3−^ anion of SDS, respectively [21,22]. The doping of SDS and HClO_4_ in PIN was verified by the presence of S=O and ClO_4_^−^ bands in FTIR spectrum [20]. Moreover, the stretching vibration of N–H bond in pyrrole ring is located as a wide band at 3112 cm^−^^1^ whereas, the presence of N–H bond suggested that nitrogen is not involved in polymerization mechanism and thus the possible polymerization sites are located at C–2 and C–3 points of pyrrole ring in the indole [19].

In the FTIR spectrum of AC, the peaks positioned at 3741 cm^−^^1^ and 1649 cm^−^^1^ are allocated to –OH of water molecules and stretching vibrations for C=O in carboxylic, aldehyde and ketones group, respectively. The peak at 1514 cm^−^^1^ is ascribed to C=C in aromatic ring stretching [23,24]. There is a broad band at 1294 cm^−^^1^ that is attributed to stretching vibration observed for C–O bond due to presence of acid, phenol, ether, esters and alcohol. Furthermore, band at 1128 cm^−^^1^ notifies the presence of groups containing phosphorous and oxygen species like symmetric vibration in P–O–P linkage and ionized linkage P^+^–O^−^ in acid phosphate ester [25]. The indication of groups composed of phosphorus species are attributed by presence of band around 1183 cm^−^^1^ due to vibration in H-bonded P=O, P–O–C stretching of O–C bond in aromatic linkage and to P=OOH [23,25].

All the characteristic peaks of PIN and AC were observed in the spectra of PIN@AC with the little effect in peak intensities and positions corresponding to varying amount of AC added. The pointed peak centered at 717 cm^−^^1^ representing out of plane deformation of C–H bond shows shift towards higher wavenumber, i.e., 727 cm^−^^1^. The Cl–O stretching band at 1036 cm^−^^1^ shows a shift to a lower wavenumber with the little increase in the broadness, but further increase in AC amount makes it to shift to 1039 cm^−^^1^. Similarly, the peak pointed at 1187 cm^−^^1^ for antisymmetric stretching of the S=O slightly slides towards lower wavenumber. The peak positioned at 1514 cm^−^^1^ is less visible, which becomes broad and more visible in composite samples with the shift towards higher wavenumber 1549 cm^−^^1^ showing the presence of aromatic ring. Upon further increase in AC amount, the peaks shifted downfield (1547 cm^−^^1^). The peak shifting to lower wavenumber region may be due to various stretching vibration of benzene ring that resulted from the interaction of AC and π-π conjugation of polymer chain [26,27]. Besides, the phosphorous containing groups’ (P–O–P, P^+^–O^−^, P=O, P–O–C, P=OOH) vibrations were also observed at 1128 cm^−^^1^ and 1187 cm^−^^1^, showing the incorporation of AC into PIN main chain. Furthermore, no extra peak was observed in composite samples except for characteristic peaks of polymer salt and AC [28,29]. The relative shift in peaks positions and presence of peaks for AC in composited samples confirms the formation of AC@PIN composite [29,30].

In addition, FTIR spectrum of AC@PIN after adsorption was also measured in order to confirm the adherence of dye with the surface (Figure 1c). Some changes in the vibrational frequencies of characteristic peaks were observed in the range of 1000–3300 cm^−^^1^ after MG adsorption. The peaks located at the 1036, 1187, 1513, and 1586 cm^−^^1^ shifted to 1039, 1189, 1538, and 1600 cm^−^^1^, respectively. These results indicate the interaction of MG with the functional groups at the surface of AC@PIN composite. Similar results were already reported in the literature [31,32].

### 3.2. Surface Morphology Analysis

SEM in conjugation with EDX spectroscopy was implemented for surface topographical, chemical composition characterization and to acquire information regarding elements present in sample as well as their percentage amounts, respectively.

**Figure 2:**
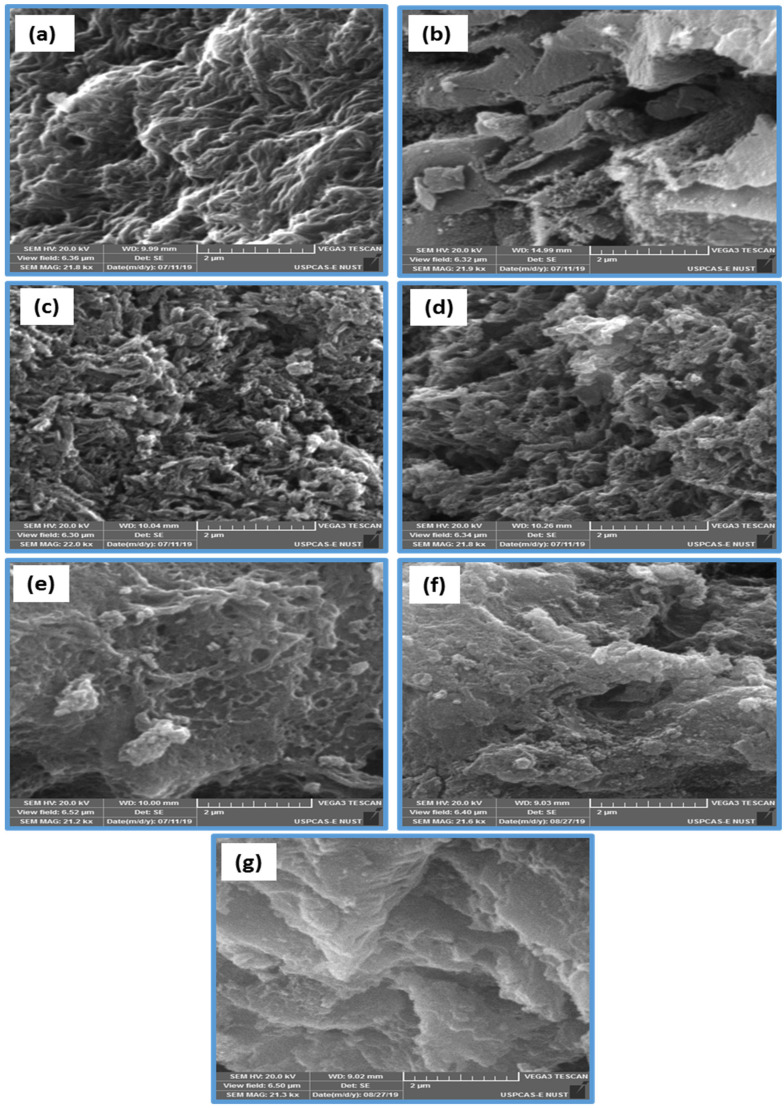
SEM micrographs of (**a**) PIN, (**b**) AC, and (**c**) AC@PIN-1, (**d**) AC@PIN-2, (**e**) AC@PIN-3, (**f**) AC@PIN-4 composite before adsorption, and (**g**) AC@PIN-3 after adsorption at the magnification of 2.0 μm.

Figure 2a depicts the SEM micrographs of PIN. It is evident that PIN has the agglomerated interlaced nano-fibrous structure and the diameter of nano-fibers measured is 33.87 nm and 35.34 nm. The agglomeration of fibers can be attributed to the presence of SDS surfactant in polymer that encompasses negative head group (being anionic surfactant) adheres well with positively charge PIN [22,33]. Figure 2b represents the SEM image of H_3_PO_4_ impregnated AC, which shows the uneven and ruptured morphology with pores or cavities on the irregular surface of AC. The removal of activating agent (H_3_PO_4_) during activation is the cause of formation of cavities [23]. The surface morphologies of PIN@AC composites are shown in Figure 2c–f. It can be seen that as the amount of AC is increased in composite sample, the nanofibers merged with each other and the interaction between AC and polymer was observed. The surface texture changes and fibers become less visible. Henceforth, the nanostructures with empty voids might be expected to exhibit good adsorptive properties facilitating easy penetration of dye molecules.Thus, it was revealed that using highest amount of AC in composite sample resulted in surface roughness and reduced porosity with disappearance of nanofibers due to dominating effect of AC over polymer fibers morphology [34,35].

Moreover, SEM micrograph of AC@PIN-3 after MG adsorption is illustrated in Figure 2g. The image shows a smooth, even, and non-porous surface after adsorption clearly indicates the interaction of composite with dye.

### 3.3. EDX Analysis

**Figure 3:**
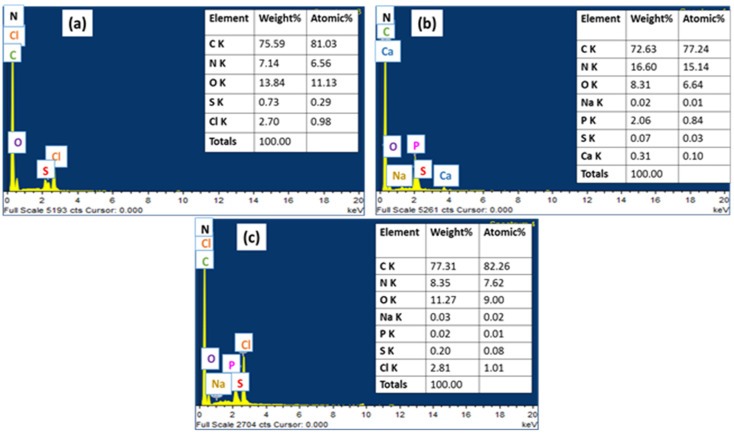
EDX spectra of (**a**) PIN, (**b**) AC, and (**c**) AC@PIN-3 composite.

The elemental composition in EDX report of pristine and AC is shown in Figure 3. The presence of C, N, O, S, and Cl revealed the successful synthesis of PIN doped with acid as well as surfactant.

The sulfur (0.73 wt.%) and Cl (2.70 wt.%) content presented the incorporation of SDS and HClO_4_, respectively. Moreover, the presence of P (2.06 wt.%) in EDX spectrum of AC confirms the chemical activation with H_3_PO_4_. However, the trace amounts of Ca and Na in EDX spectrum of AC shows might be referred to be coming from the precursor used for AC preparation. Furthermore, we obtained the elemental compositions of AC@PIN composite (Figure 3c). The data of elemental analysis showed all the characteristic elements observed in EDX spectra of PIN and AC. The carbon content is increased to 77.31 (wt.%) in AC@PIN-3, which confirms the integration of AC with PIN matrix.

### 3.4. X-ray Diffraction Analysis

**Figure 4:**
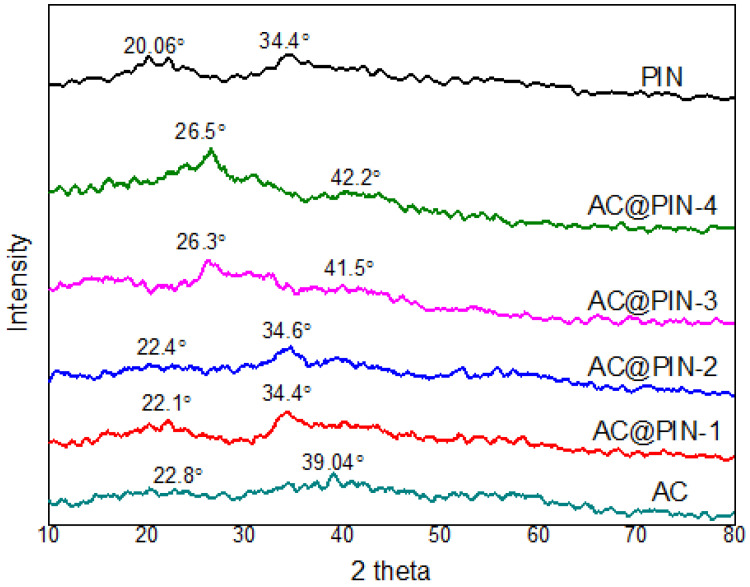
Comparison of XRD pattern of PIN and PIN@AC composites.

XRD analysis of AC shown in Figure 4 depicts two characteristic diffraction peaks for AC centered at 2θ values of 22.8° and 39.04°. The XRD plot revealed amorphous nature of AC with the very little percentage of crystallinity that is consistent with previous researches. The ordered crystalline phase is negligible, which leads to amorphous nature of AC [36]. The presence of third pointed peak in the sample may arise due to leftover or excess ash in carbon sample [37].

The XRD spectrum of PIN exhibited peaks at 20.06° and 34.44° (Figure 4). The broad peak confirms the indole polymerization. These peaks confirmed amorphous nature of the synthesized polymer [38,39]. The XRD pattern recorded for the AC@PIN composites showed similar characteristic increase in 2theta values for AC@PIN composites. Hence, the shift in peaks positions to right and increase in peak broadness is attributed to the presence of AC which confirms the formation of AC@PIN composite [40]. Thus, characteristic peaks of polymer are less visible which on addition of AC in composite resulted in domination of peaks for AC with increase intensity.

### 3.5. UV-Visible Spectroscopy

**Figure 5:**
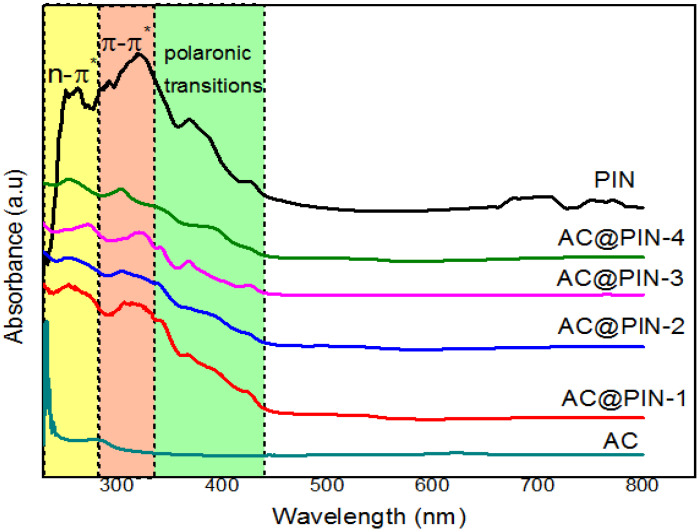
UV-Vis spectra of PIN, AC, and AC@PIN composites.

UV-Vis linear absorption spectra for PIN, AC, and AC@PIN composites that were dissolved in ethanol are shown in Figure 5. For PIN, numerous peaks appeared. Peaks at 254 nm originates from n-π* transition, whereas peaks positioned at 325 nm and 388 nm are due to the π-π* transition. Moreover, absorption peak at 428 nm and 505 nm arises due to polaronic transition in PIN [41,42]. Compared to indole UV-Vis spectrum as in the literature, the presence of more peaks with their shift towards the longer wavelength (bathochromic shift) in the spectra of PIN provides the evidence of higher extent of π-electronic conjugation in polymer. The enhanced π-electronic conjugation results in more π-bonds and thus improvement in delocalization of electron along pi-bond of PIN synthesized is recorded [43,44]. On the other hand, UV-Vis spectrum of AC shows a sharp and a broad band below 35 nm, which represents n-π* and π-π* transitions, respectively.

Figure 5 shows UV-Vis spectra of AC@PIN composites. From the spectra, it is evident that with increasing AC amount, a change in band intensity was observed compared to PIN doped with only HClO_4_ and surfactant SDS. With increasing AC content, the intensities for the peaks associated with π-π* transition was decreased and shifted to longer wavelength. Absorbance at 325 nm shifted to 343 nm and the peak positioned at 389 nm for polymer sample shifted to 397 nm with rising AC amount up to 0.25 g. However, on further addition of AC beyond 0.24957 g cause a reduction (391 nm) in wavelength. Moreover, third peak was found to experience little shift from 424 nm to 428 nm with addition of AC. The decrease in intensity of peak was observed, which may be attributed to decrease in conjugation of PIN due to composite formation with AC [35,45].

### 3.6. Point of Zero Charge (pH_PZC_)

**Figure 6:**
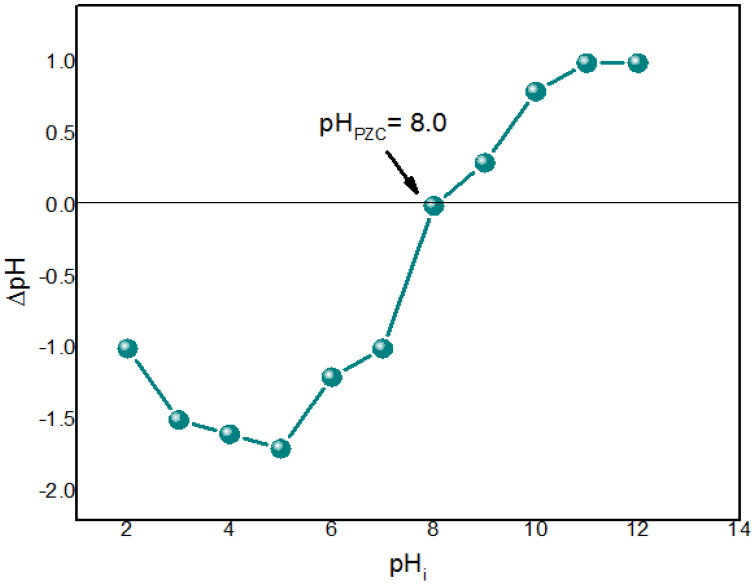
Point of zero charge (pH_PZC_) of AC@PIN composites.

The adsorption capacity of AC@PIN composite and the nature of binding sites were determined from the point of zero charge (pH_PZC_). When the pH is less than pH_PZC_, the surface of adsorbent is expected to be negatively charged and it allows adsorption of positive ions. At pH greater than pH_PZC_, the adsorption of anions is more favorable due to increased positive ions at the adsorbent surface [8]. As shown in Figure 6, the pH_PZC_ for AC@PIN composite is 8.0. Thus, it can be inferred that protonation of surface functional groups of AC@PIN occurred below pH_PZC_and a strong electrostatic forces of attraction was developed between the anions of MG with the surface positive charges. Conversely, the negatively charged groups dominated the AC@PIN surface at pH greater than pH_PZC_. Therefore, adsorption of cationic dye, MG, was favored above pH = 8.0.

### 3.7. Adsorption Studies

#### 3.7.1. Adsorption Efficiency of Prepared PIN, AC, and AC@PIN

**Figure 7:**
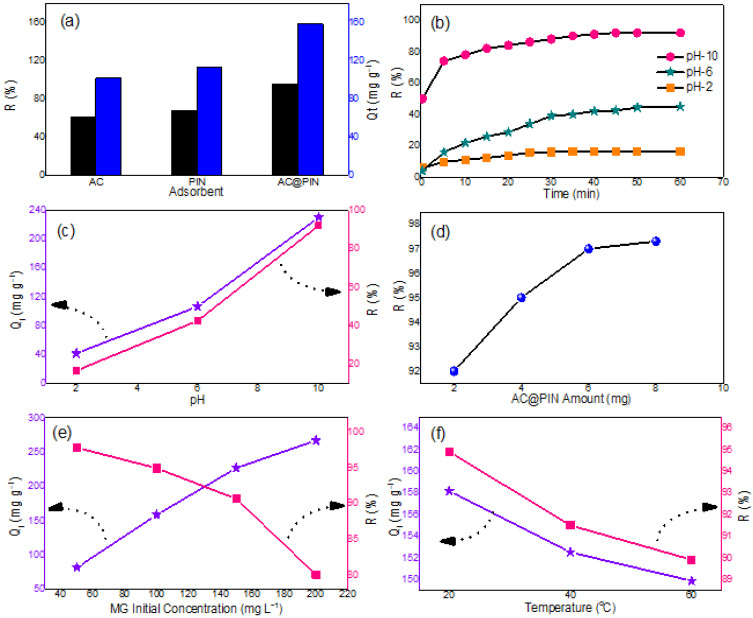
(**a**) Adsorption efficiency and capacity of AC, PIN, and AC@PIN composite, (**b**,**c**) percent removal of MG as a function of time and pH [Co = 50 mg L^−1^; Temperature = 20 °C; AC@PIN = 2 mg], (**d**) effect of AC@PIN amount on adsorption of MG [Co = 50 mg L^−1^; Temperature = 20 °C; pH = 10; contact time = 60 min], (**e**) effect of initial concentration on MG adsorption [Temperature = 20 °C; pH = 10; contact time = 60 min; AC@PIN = 6 mg], and (**f**) effect of temperature on adsorption of MG [Co = 100 mg L^−1^; pH = 10; contact time = 60 min; AC@PIN = 6 mg].

Figure 7a shows the adsorption efficiency of the three prepared adsorbent materials for the removal of MG from aqueous solution. It is clear from the graph that the adsorption efficiency of AC, PIN, and AC@PIN composite was 60.9%,68.0%, and 94.9%, respectively. The maximum MG uptake of 101.5, 113.3, and 158.2 mg g^−^^1^ was attained by AC, PIN, and AC@PIN composite, respectively. These results revealed that the adsorption properties of the prepared AC@PIN composite have been greatly enhanced due to the integration of the two components (AC and PIN). Therefore, we selected AC@PIN composite as better adsorbent material for MG removal. The detailed investigation of adsorptive behavior of AC@PIN was carried out and the results are discussed below.

#### 3.7.2. Influence of pH and Contact Time

To understand the adsorption process, the study of various factors effecting the adsorbent/solution interface needs to be evaluated. One of the major factor that influences the adsorption phenomenon is solution pH as it affects the adsorbent surface charge, ionization state, and solubility of dye [17,32]. Therefore, we carried out the adsorption of MG on the AC@PIN composite as a function of pH ranging from 2 to 10 at 20 °C. The dye initial concentration was 50 mg L^−^^1^. The effects of pH on the adsorptive removal of MG by AC@PIN composite are presented in Figure 7b,c. It can be seen from the figure that the percent removal of MG was 16.4% and 42% at pH = 2 and 6, respectively. This can be attributed to the positively charged surface of the AC@PIN composite at pH < pH_PZC_, which is less favorable for interaction with cations of MG. It is expected that the surface might adsorb H^+^ ions from aqueous system, becomes positively charged, and cause electrostatic repulsion between the AC@PIN surface and MG cations. Hence, it results in the less adsorption of MG from solution in acidic pH [2,46]. On the other hand, a marked increase in the percent removal of MG adsorption was observed as the pH is increased from 6 to 10. Interestingly, around 92% of MG was adsorbed from the solution which is ascribed to the deprotonation of the AC@PIN composite surface resulting in the more negative charges at pH > pH_PZC_. Therefore, the electrostatic attraction between the composite and MG cations increases, which facilitates the removal of MG. Based on these results, it can be concluded that the alkaline pH favored MG adsorption, therefore, further adsorption experiments were performed at pH = 10 [31,46].

The dye contact time with AC@PIN composite determines the optimum time required for adsorption process to attain equilibrium [17]. The adsorption of MG from aqueous solution at different time intervals (0–60 min) and at various pH (2–10) was investigated and the results are displayed in Figure 7b. In the initial 10–15 min of contact with the AC@PIN composite, the adsorption of MG is fast, and then, it decreases as equilibrium point is reached in around 40 min at each pH. This is ascribed to the fact that a large number of adsorption active sites are accessible to MG in the start of contact. With the passage of time, the dye cations accumulated at the surface of AC@PIN composite, which restricts the other ions to penetrate deep into the adsorbent molecular structure. Hence, the adsorption rate is reduced and equilibrium situation is achieved. These results are consistent with the previous reports [4,17,32]. Based on these results, 60 min contact time was considered as the optimum equilibrium time throughout the experiments.

#### 3.7.3. Influence of AC@PIN Dosage

The effect of AC@PIN dosage on the adsorption of MG was investigated in the adsorbent dosage range of 2.0 to 8.0 mg. Figure 7d illustrates that the adsorption efficiency is greatly dependent on the AC@PIN amount. The percent removal of MG increased as the amount of AC@PIN was increased. This observation is better explained by the availability of large number of accessible active sites of the AC@PIN composite [1,4]. The maximum value of MG removal efficiency of about 97% was removed by 6.0 mg AC@PIN composite. Further increase in adsorbent dosage did not show any significant increase in adsorption efficiency. This might be due to the possible overlapping of the binding sites due to aggregation of adsorbent particles under high dosage of the composite. Similar results are also reported by Abdelghani et al. [1]. They noticed similar behavior of PANI@AS composite towards Orange G dye adsorption. Thus, 6.0 mg was fixed as the optimal adsorbent dosage for further experiments.

#### 3.7.4. Influence of MG Concentration

The dye concentration is another important parameter to investigate adsorption process. The influence of MG initial concentration on the adsorption uptake and removal efficiency of AC@PIN was evaluated by varying initial dye concentration from 50 to 200 mg L^−1^. As shown in Figure 7e, the adsorption capacity increased from 81.5 to 266.6 mg g^−1^ with the increase in initial MG concentration. The adsorption capacity of AC@PIN composite is low under low concentration condition of MG which is due to the weak driving force rendering less number of collisions between MG molecules. This results in low mass transfer from liquid to phase to the adsorbent surface [2]. At high initial concentration of MG, the adsorption capacity is high. This might be due to the increased collisions between MG molecules resulting in high driving force and low resistance, thus, promoting a large mass transfer from liquid to solid. The surface active sites are completely saturated with MG under high initial concentration of MG [4,32]. Moreover, the decrease in dye removal efficiency (from 97.8 to 80%) with increase in MG concentration is clearly evident from Figure 7e. This can be attributed to the unavailability of active binding sites on AC@PIN surface. These results coincide with previous reports [2,32].

#### 3.7.5. Influence of Temperature

Finally, the effect of temperature on the adsorption of MG onto AC@PIN was investigated by varying the temperature from 20 to 60 °C. Figure 7f illustrates the adsorption capacity and percent removal of MG by AC@PIN composite as a function of temperature. It can be clearly seen from the graph that the adsorption capacity and removal efficiency decreased with the increase in temperature. This revealed the exothermic nature of MG adsorption onto the composite. The increased kinetic energy and mobility of MG ions with the rise in temperature might have caused the reduction in adsorption [2,32].

#### 3.7.6. Adsorption Kinetics

**Figure 8:**
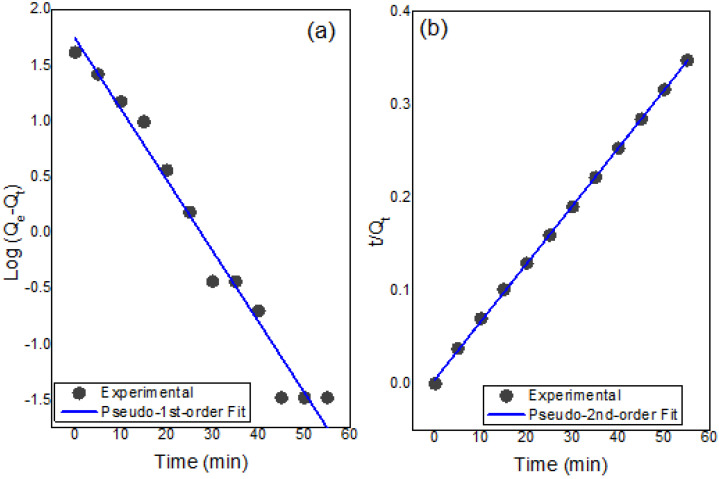
Plots of the (**a**) pseudo-1st-order, and (**b**) pseudo-2nd-order model for MG adsorption onto AC@PIN composite [Co = 150 mg L^−1^; Temperature = 20 °C; AC@PIN = 6 mg, contact time = 60 min, pH = 10].

The kinetic study of adsorption process is important to select the optimum operating parameters for the complete adsorption process. Kinetic study is helpful to gain information about the rate, reaction pathway, diffusion, and mass transfer mechanism of dye adsorption [4,32]. To evaluate the kinetics of MG adsorption on AC@PIN composite, pseudo-first-order and pseudo-second-order model were employed. The linear expression of pseudo-1st-order and pseudo-2nd-order kinetic models is given in Equations (3) and (4), respectively [4,9].

log [Q_e_ − Q_t_] = log Q_e_− [k_1_/2.303]t (3)

t/Q_t_ = 1/[k_2_ Q_e_^2^] + t/Q_e_ (4)

where Q_e_ (mg g^−1^) is the adsorption capacity at equilibrium, Q_t_ (mg g^−1^) is the adsorption capacity at time t (min), k_1_ (min^−1^) is the pseudo-1st-order rate constant, and k_2_ (g mg^−1^ min^−1^) is the pseudo-2nd-order rate constant. The kinetic models linear fitting to experimental data points of MG adsorption onto AC@PIN composites are displayed in Figure 8a,b and the values of fitting parameters for respective models are given in Table 1. The regression coefficient (R^2^) values of the pseudo-1st-order and pseudo-2nd-order model are 0.97 and 0.99, respectively. Moreover, the experimental Q_e_ value is in close agreement with the calculated Q_e_ from pseudo-2nd-order model. These findings indicated that pseudo-2nd-order model is best fitted to our adsorption experimental data.

#### 3.7.7. Adsorption Isotherms

The isotherm modelling is a helpful tool to understand the adsorbent surface properties, distribution of adsorbate molecules at the interface, and interaction between adsorbent and adsorbate [1,2,46]. Langmuir and Freundlich isotherm models are the most commonly employed adsorption isotherm models to a solid–liquid system. Langmuir isotherm model predicts the monolayer adsorption on a homogeneous adsorbent surface where finite number of active sites are present. Besides, it assumes that no interaction between adsorbed molecules occurs on the surface. The linear expression of Langmuir isotherm model is given in Equation (5) [2,32].

Ce/Q_e_ = [Ce/Q_m_] + 1/[K_L_ × Q_m_] (5)

where Q_e_ (mg g^−1^) is adsorption capacity and Ce (mg L^−1^) is MG concentration in equilibrium condition. K_L_ is Langmuir isotherm constants and it represents the affinity of active sites and Q_m_ (mg g^−1^) is the monolayer adsorption capacity.

The validity of Langmuir isotherm model is verified from the Langmuir separation factor (R_L_). R_L_ is related to the favorability of the adsorption process. If R_L_ value is between 0 and 1, the adsorption process is favorable, while the value of R_L_ great than 1 indicates unfavorable adsorption. The following Equation (6) is used to determine R_L_ [2].

R_L_ = 1/[1 + K_L_ × C_o_] (6)

**Figure 9:**
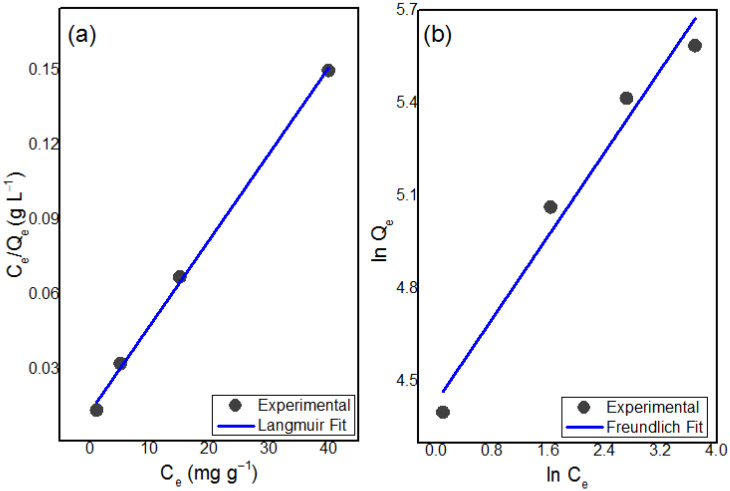
Isotherm model plots for MG adsorption onto AC@PIN composite (**a**) Langmuir and (**b**) Freundlich isotherms [AC@PIN = 6 mg; Temperature = 20 °C; contact time = 60 min; pH = 10].

Freundlich isotherm model predicts multilayer adsorption process on a heterogeneous surface. It assumes the possible interaction between adsorbate and adsorbent surface with the uneven distribution of active sites. The linear form of Freundlich isotherm model is in Equation (7) [2,32].

ln Q_e_ = ln K_F_ + [1/n] ln C_e_ (7)

where K_F_ (mg g^−1^) is Freundlich isotherm constant which express the extent of adsorption, C_e_ (mg L^−1^) is dye equilibrium concentration, Q_e_ (mg g^−1^) is equilibrium adsorption capacity and n (g L^−1^) is Freundlich isotherm constant which is related to the adsorption intensity. The linear plot of Langmuir and Freundlich isotherm models are presented in Figure 9 and parameter values are listed in Table 2.

It can be clearly seen from Figure 9 and Table 2 that the adsorption experimental data fitted well in Langmuir isotherm model with the R^2^ value of 0.99. The good correlation between experimental Q_e_ (226.5 mg g^−1^) and calculated Q_e_ (289.01 mg g^−1^) further suggests that Langmuir isotherm model is better fitted than Freundlich model (R^2^ = 0.95) for the MG adsorption onto AC@PIN. The viability of Langmuir isotherm models is also confirmed from the value of R_L_ (0.07 > 0). In addition, the value of n (2.9 > 1) implies that MG adsorption onto AC@PIN is favorable.

#### 3.7.8. Thermodynamic Study

To get the better understanding of MG adsorption on AC@PIN composite, the thermodynamic parameters including entropy change (∆S°), enthalpy change (∆H°), and Gibbs free energy change (∆G°) are computed in this study using the given Equations (8)–(10) [9,32].

∆G° = −RT ln K_c_ (8)

where K_c_ is the adsorption equilibrium constant which is calculated as;

K_c_ = C_ae_/C_e_ (9)

where C_ae_ (mg L^−1^) is the dye equilibrium concentration at the adsorbent surface, while C_e_ (mg L^−1^) is the dye equilibrium concentration in solution.

The values of ∆H° and ∆S° of the adsorption process is estimated from slope and intercept of the plot (ln K_c_ vs. 1/T) obtained through Van’t Hoff Equation (10).

ln K_c_ = [−∆H°/RT] + [∆S°/R] (10)

**Figure 10:**
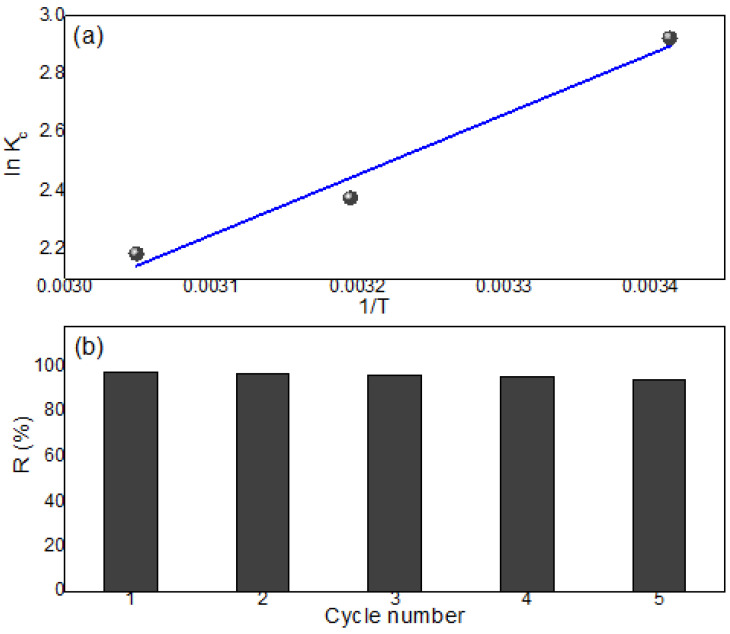
(**a**) Van’t Hoff plot and (**b**) reusability test of AC@PIN composite.

The thermodynamic parameters were computed graphically from Figure 10a and are tabulated in Table 3. The feasibility and spontaneity of the MG adsorption on the prepared composite is clearly indicated by the negative values of ∆G°. Moreover, ∆G° values increases as the temperature is raised from 298–328 K. This shows that the MG adsorption is more favorable at high temperature [17,32]. Besides, ∆H° value is negative and is less than 40 kJ mol^−1^. This revealed the exothermic and physical adsorption of MG on the AC@PIN. The negative value of ∆S° confirmed that no significant changes occur in the internal structure of AC@PIN [9,32].

#### 3.7.9. Reusability Study

The practical implementation of the prepared adsorbent is assessed by the reusability test. Therefore, we subjected the AC@PIN to desorption–adsorption for five cycles. From Figure 10b, it is evident that the prepared AC@PIN composite has excellent regeneration capacity even after five desorption–adsorption cycles. The relatively small decrease in the removal efficiency from 97.8% (1st cycle) to 94.4% (5th cycle) is observed due to the incomplete desorption of AC@PIN [4].

#### 3.7.10. Probable Mechanism of Adsorption

**Scheme 3:**
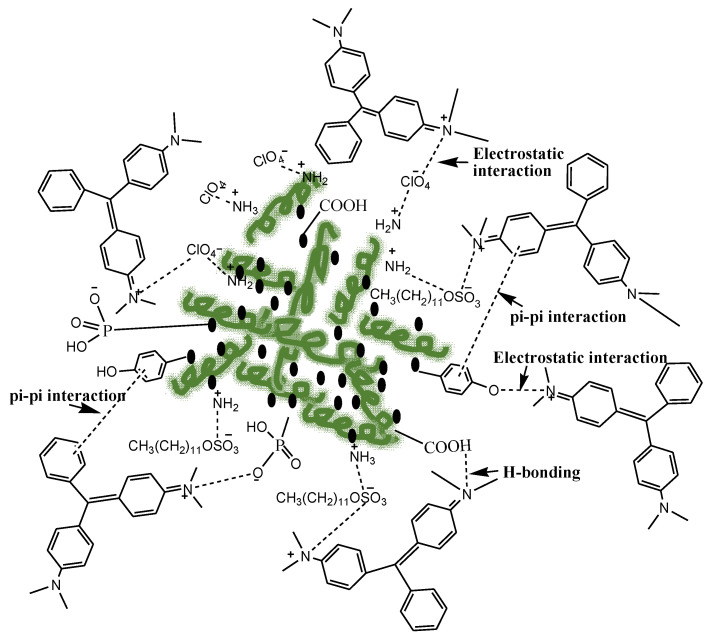
Possible adsorption mechanism of MG onto AC@PIN.

The adsorption process is majorly dependent on the nature of adsorbate and surface chemistry of adsorbent. The proposed mechanism of MG adsorption onto AC@PIN is presented in Scheme 3. It can be proposed that the MG adsorption onto AC@PIN involves π-π interactions, electrostatic interactions, and hydrogen bonding. From the FTIR results, it is clear that the surface of AC@PIN composite is negatively charged due to the presence of ClO_4_^−^, CH_3_(CH_2_)_10_SO_3_^−^, HPO_3_^−^, C_6_H_5_OH, and COOH functional groups. At pH = 10, greater than pH_PZC_, it is expected that the electrostatic interaction is more dominant between negatively charged species of AC@PIN and positively charged nitrogen species of MG in solution. Moreover, the hydrogen bonding between surface hydrogen containing groups and N atom of MG dyes as well as the π-π interaction between π-electrons of the benzene rings in AC@PIN and MG also plays a dominant role.

Table 4 compares various polymer-based adsorbents reported previously for the removal of MG. The Q_e_ (226.5 mg g^−1^) value of AC@PIN composite prepared in the current work is comparable to other adsorbents. Besides, AC@PIN is relatively more efficient, eco-friendly, and easy to prepare in bulk amounts compared to the corresponding ones.

## 4. Conclusions

AC@PIN composites were synthesized via a facile and convenient in situ chemical polymerization method, and the same was employed for the adsorptive removal of Malachite Green dye from aqueous solution. The prepared composites were characterized by FTIR, SEM, XRD, EDX, and UV-Vis spectroscopy. The process variables (pH, contact time, AC@PIN amount, Malachite Green initial concentration, and temperature) were found to have a significant influence on the MG adsorption on the AC@PIN. The maximum adsorption capacity of 226.5 mg g^−1^ and 97.3% removal efficiency was attained at pH = 10 in the contact time of 60 min at temperature 20 °C. Moreover, the pseudo-2nd-order kinetic model and Langmuir isotherm model fitted well to the MG adsorption on the prepared composite. In addition, the adsorption of dye was found to be exothermic in nature. Though, the adsorption capacity is not much higher, still the prepared AC@PIN composite is comparatively more efficient as it can remove 97.3% of the dye from aqueous system by attain equilibrium in relatively less time. Besides, it is a novel and an eco-friendly adsorbent which can be prepared at low cost in large amount by a simple and convenient one-pot chemical oxidation method. Overall, the promising adsorption efficiency makes the prepared AC@PIN composite as a potential contender in the list of available adsorbents for the removal of dye from wastewater.

## Figures and Tables

**Table 1:** Kinetic parameter values for the MG adsorption on AC@PIN composite.

Experimental	Pseudo-1st-Order			Pseudo-2nd-Order		
Q_e_ (mg g^−1^)	Q_e_ (mg g^−1^)	K_1_ (min^−1^)	R^2^	Q_e_ (mg g^−1^)	K_2_ (g mg^−1^ min^−1^)	R^2^
226.5	51.12	−1.46	0.97	250	0.003	0.99

**Table 2:** Isotherm parameter values for adsorption of MG on AC@PIN composite at 20 °C.

Experimental	Langmuir Isotherm Model			Freundlich Isotherm Model		
Q_e_ (mg g^−1^)	Q_e_ (mg g^−1^)	K_L_	R^2^	K_F_ (mg g^−1^)	n	R^2^
226.5	289.01	0.271	0.99	26915	2.9	0.95

**Table 3:** Calculated values of thermodynamic parameters.

Temperature (K)	∆G° (kJ mol^−1^)	∆H° (kJ mol^−1^)	∆S° (kJ mol^−1^ K^−1^)	R^2^
293	−27.23	−17.15	−0.034	0.95
313	−27.92			
328	−28.43			

**Table 4:** Comparison of adsorption properties of various polymer based adsorbents towards Malachite Green dye.

Adsorbents	Q_e_ (mg g^−1^)	Equilibrium Time (min)	Adsorbent Dosage (g)	Kinetic Study	Isotherm Study	Thermodynamic Study	Ref
PVA/CMC/SA/ZSM ^a^	5.95	220	5.0	Pseudo-2nd-order	Freundlich	-	[2]
Fe_2_O_3_@PANI	240.0	240	0.01	Pseudo-2nd-order	Langmuir	exothermic	[47]
PANI-nickel ferrite	4.09	210	5.0	Pseudo-2nd-order	Langmuir	-	[48]
Poly(methyl methacrylate)/graphene oxide-Fe_3_O	3.5	35	0.005	Pseudo-2nd-order	-	exothermic	[49]
Kappa-carrageenan-g-polyacrylic acid/TiO_2_-NH_2_	666.66	180	0.05	Pseudo-2nd-order	Langmuir	exothermic	[50]
Poly(vinyl alcohol)-chitosan	380.6	540	0.1	Pseudo-2nd-order	Freundlich	endothermic	[51]
AC@PIN	226.5	60	0.006	Pseudo-2nd-order	Langmuir	exothermic	**This work**

^a^ = polyvinyl alcohol-carboxymethyl cellulose-sodium alginate zeolite-5.

## Data Availability

The data presented in this study are available on request from the corresponding authors.
